# Disruption of RNA metabolism and its impact on protein homeostasis

**DOI:** 10.1098/rstb.2025.0267

**Published:** 2026-10-01

**Authors:** Susana M. D. A. Garcia

**Affiliations:** Institute of Biotechnology, HiLIFE, University of Helsinki, Helsinki, Finland

**Keywords:** RNA repeat toxicity, energy metabolism, mitochondria, *Caenorhabditis elegans*, proteostasis, myotonic dystrophy

## Abstract

Repeat-associated disorders arise from expansions of nucleotide repeats in coding or non-coding regions, producing RNAs that accumulate and disrupt cellular homeostasis through mechanisms collectively termed RNA repeat toxicity. This process complements proteotoxicity in coding-region expansions and is increasingly recognized as a major contributor to pathogenesis. Expanded repeat RNAs form stable secondary structures that sequester RNA-binding proteins (RBPs), such as Muscleblind-like, alter splicing and activate small RNA pathways, broadly impacting gene expression. Myotonic dystrophy type 1 (DM1) exemplifies RNA-mediated toxicity, with expanded CUG repeats in the *DMPK* 3′ untranslated region driving nuclear foci formation, RBP sequestration and transcriptome-wide changes. Beyond RNA processing defects, DM1 exhibits systemic dysfunction, notably affecting energy metabolism and mitochondrial function, which recent evidence suggests may be disrupted early and progressively during disease development. These metabolic changes intersect with proteostasis failure, amplifying cellular stress and contributing to systemic pathology. A deeper understanding of the molecular networks that drive metabolic and mitochondrial dysfunction, and their role in proteostasis collapse, will be critical for elucidating disease onset and progression. This knowledge will enable the identification of novel therapeutic targets and biomarkers, offering strategies that go beyond RNA-centric approaches to restore cellular homeostasis in repeat-based disorders.

This article is part of the Theo Murphy meeting issue ‘ProteostaSys: a systems view of proteostasis’.

## 1. Genomic repeat expansions and their role in disease pathogenesis

Nucleotide repeat sequences are found in both prokaryotic and eukaryotic organisms and are particularly abundant in mammals [1]. Current estimates indicate that repetitive sequences account for approximately half to two-thirds of the genome [2]. These elements are not merely passive; they have been implicated in gene expression regulation, genetic diversity and evolutionary processes [1]. Repeats occur in both coding and non-coding regions, with notable enrichment in introns [1]. Owing to their repetitive nature, these sequences can adopt complex secondary and tertiary structures that interfere with replication, transcription and DNA repair [3], contributing to genomic instability and a high mutation rate. Changes in nucleotide repeat length resulting in expansions have so far been associated with more than 50 neuromuscular degenerative disorders [4,5].

Repeat-based disorders include well-known diseases, such as Huntington's disease (HD), amyotrophic lateral sclerosis, myotonic dystrophies (DMs) and several spinocerebellar ataxias (SCAs) [4]. Decades of research aimed at understanding the pathogenic mechanisms underlying repeat-based toxicity have led to the development of multiple conceptual models. For disorders involving triplet repeat expansions, pathogenesis is broadly categorized into two mechanistic classes: loss-of-function and gain-of-function.

Loss-of-function may result from gene haploinsufficiency owing to epigenetic silencing or impaired protein function caused by inserted polypeptide tracts [6,7]. Gain-of-function mechanisms arise when the presence of expanded repeats confers new toxic properties to RNA and/or protein. At the RNA level, transcripts bearing expanded repeats can aberrantly bind RNA-binding proteins (RBPs), disrupting their function (impairing or activating) [8–12]. Additionally, expanded repeats can undergo non-canonical, repeat-associated non-AUG (RAN) translation, producing low-complexity toxic peptides [13]. These toxic peptides can accumulate in the cell and have been linked to several degenerative disorders including HD, SCAs and DMs [14,15]. At the protein level, expanded repeats in coding regions generate repetitive polypeptides, such as polyglutamine (polyQ) tracts in mutant huntingtin, which misfold, aggregate, impair degradation and ultimately disrupt proteostasis [16]. While protein-based mechanisms have long dominated research on repeat disorders, growing evidence highlights RNA repeat toxicity as an equally critical contributor to pathogenesis, warranting focused investigation.

## 2. RNA repeat toxicity as a common pathogenic mechanism in repeat-associated disorders

Transcription of genomic loci containing expanded repeats, whether in coding regions or non-coding regions, such as introns and 3′ untranslated regions (3′UTRs), generates mutant RNAs that contain these pathogenic expansions. The expression and accumulation of these RNAs can disrupt cellular homeostasis, and over the past decades, RNA repeat toxicity has emerged as a key contributor to disease pathogenesis, independent of the genomic location of the repeat.

Early evidence for this mechanism came from a seminal study by Li *et al.* [17] using a *Drosophila* model of SCA type 3 (SCA3). SCA3 is caused by a CAG repeat expansion in the coding region of the *MJD* gene, producing a protein with a polyQ tract. To distinguish RNA from protein toxicity, the authors engineered a truncated *MJD* gene (MJDtr) in which pure CAG repeats were replaced with interrupted CAACAG repeats. This modification preserved the polyQ protein sequence but eliminated pure repeats in the RNA. Remarkably, this change dramatically reduced toxicity, demonstrating that RNA repeat toxicity is a key pathogenic factor alongside proteotoxicity [17].

Mechanistically, RNAs with expanded CNGs adopt stable hairpin structures that can engage specific RBPs. These include the Muscleblind-like (MBNL) family and the RNases Dicer and Drosha, which recognize CAG, CUG and CCG repeat sequences as common binding targets [10,18–21]. Sequestration of MBNL proteins by expanded repeat RNAs depletes their functional pool, broadly altering RNA processing, such as splicing and transcriptome organization [11]. Conversely, Dicer binding to repeat RNAs activates the siRNA pathway, generating short silencing RNAs that target endogenous transcripts containing complementary CNG motifs, thereby perturbing gene expression. Dicer is a riboendonuclease best known for its central role in the small RNA pathways (microRNAs and RNA interference pathways), although it also participates in several other cellular processes.

Genetic screens for modifiers of repeat-based pathogenesis, caused by either expanded protein or RNA repeat toxicity, have identified not only RBPs but also factors involved in protein folding and ubiquitin-mediated degradation [22–24]. These findings underscore shared pathogenic pathways between toxic polyQ proteins and expanded repeat RNAs (e.g. CAG and CUG), highlighting RNA repeat toxicity as a central mechanism in repeat-associated diseases.

## 3. Expanded CUG repeats and myotonic dystrophy: a model for RNA repeat pathogenesis

Myotonic dystrophy type 1 (DM1) was the first disorder identified as being caused by expanded RNA repeats in 1992 [25–29] and remains a widely used model for studying RNA-mediated pathogenesis [30]. DM1 is the most common form of muscular dystrophy in adults and is caused by an expansion of CTG repeats in the 3′UTR of the *Dystrophia Myotonica-Protein Kinase* (*DMPK)* gene [31]. DMPK encodes a serine/threonine kinase proposed to participate in several biological processes [32] and known to interact with proteins, such as Lamin-A/C, and chaperones, such as HSPB2 [33,34]; however, its precise physiological functions remain poorly understood.

Clinically, DM1 is an autosomal dominant, multi-systemic disorder characterized by progressive muscle weakness, atrophy and myotonia [31,35]. In DM1-affected individuals, CTG repeat lengths range from 50 to 4500, whereas healthy individuals carry 5–35 repeats [36]. Repeat sizes of 37–50 define a premutation state, which is generally asymptomatic [37].

Repeats are inherently unstable, and their length can change spontaneously owing to errors in DNA replication, repair and recombination during germline transmission [38]. These processes generate substantial intergenerational variability in *DMPK* repeat length. Once CTG repeat sizes exceed the pathogenic threshold, the expanded alleles become markedly more prone to further instability than short, wild-type alleles [39]. This heightened instability gives rise to the phenomenon of genetic anticipation in DM1 patients, characterized by progressive repeat expansion across generations [40]. As in other repeat-based disorders, expansion length correlates with disease onset and severity [41,42], although somatic instability and sequence interruptions can significantly modify this relationship [43,44].

At the molecular level, RNAs with expanded CUG repeats accumulate in the nucleus, form foci and drive gain-of-function mechanisms that disrupt cellular homeostasis and promote degeneration [45] (figure 1). These RNA foci, also referred to as nuclear RNA condensates, arise when expanded-repeat RNAs fold into stable hairpins and assemble into higher-order complexes. Although still not fully understood, these condensates consist of repeat-expanded RNAs that engage in extensive RNA–RNA intermolecular interactions [46] together with RBPs, most notably MBNL proteins [47]. Recent studies indicate that, in addition to trans RNA–RNA interactions, proteins, such as MBNL, substantially influence foci formation by contributing to their stability, size and number [47]. MBNL proteins may act as crosslinkers that promote the formation of larger RNA clusters [47]. Other RBPs also associate with expanded RNAs, but their specific roles in foci biogenesis remain unknown. Clarifying how these proteins modulate condensate formation and function will be an important direction for future research.

**Figure 1.**
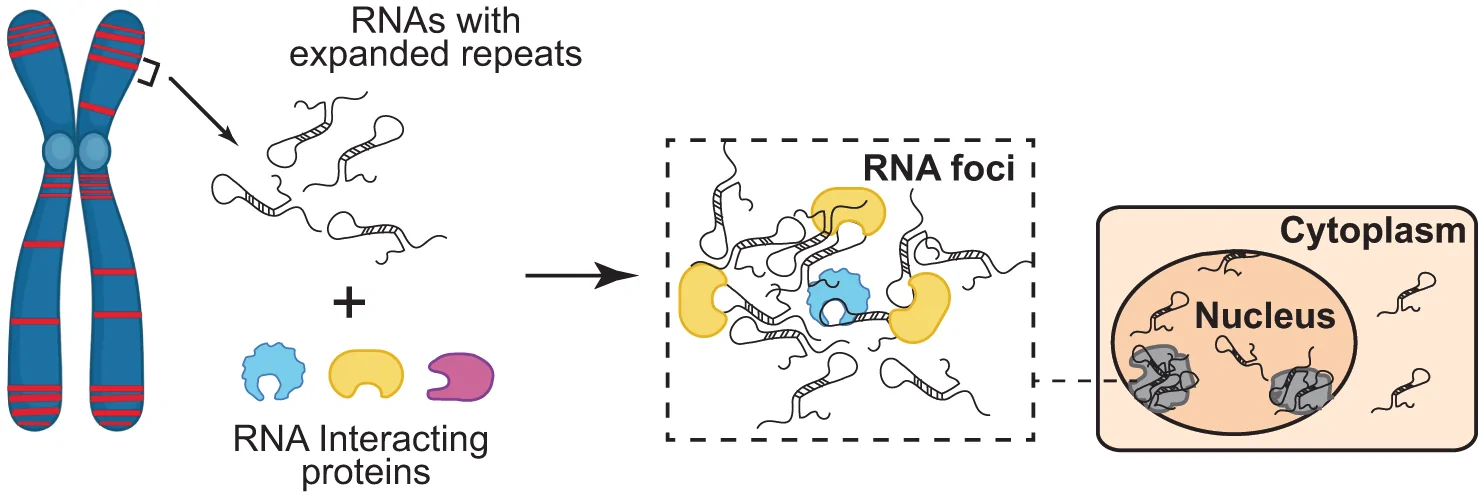
Model of recruitment and sequestration of RBPs by RNAs with expanded repeats. Transcripts with expanded repeats accumulate in the nucleus as foci.

Nuclear foci, first observed in DM1 patient samples, are considered a hallmark of RNA repeat disorders [48]. Their number and size correlate with repeat length and disease onset [22]. Multiple mechanisms are proposed to underlie CUG RNA toxicity, with pathogenesis expected to result from a combination of several toxic processes. Still, RBPs sequestration is regarded as a central driver owing to its widespread impact on RNA expression and processing. Among these, the developmentally regulated RBP MBNL is the best-characterized modifier of CUG RNA toxicity [11] and is widely considered a key contributor to DM1 pathogenesis. MBNL proteins recognize the RNA motif YGCY (Y = U or C) [49], and thus bind and are retained by RNAs with expanded CUGs [50,51]. Because MBNLs regulate multiple aspects of RNA metabolism—alternative splicing [52,53] and polyadenylation [54], subcellular localization [53,55] and mRNA stability [53,56]—their functional loss leads to extensive transcriptome disruption, making it a focal point for mechanistic studies and therapeutic strategies [57].

In addition to MBNL, other modifiers of CUG RNA toxicity have also been identified—including RBPs, such as RBFOX1-3, CELF1 (CUGBP1), RNA helicases DDX5 and DDX6, as well as Dicer, Staufen1 and hnRNPs (hnRNP H, hnRNP L and hnRNP A1) [58,59]. However, the precise mechanisms driving cellular dysfunction and promoting disease progression remain poorly understood. Additional factors beyond RBPs are likely to play significant roles in promoting cellular toxicity.

Together, these findings illustrate that RNA-mediated mechanisms—particularly RBP sequestration and the involvement of additional RNA-processing factors—represent major contributors to cellular dysfunction in DM1. Nevertheless, how these mechanisms give rise to the full range of molecular and physiological abnormalities observed in patients remains unclear. DM1 and related RNA repeat disorders are multi-systemic. In addition to skeletal muscle degeneration, DM1 pathology affects respiratory, endocrine and central nervous systems [60–62], and disrupts diverse molecular pathways, including signalling, transcription, RNA silencing and energy metabolism [35,63,64]. Understanding which pathways are most critical for disease progression, and when these disruptions occur during development, remains a major research goal. Among these, energy metabolism stands out as an early and potentially underestimated contributor to pathogenesis, which we explore in the following sections.

## 4. Metabolic dysfunction in RNA repeat disorders: insights from Myotonic dystrophy type 1

Energy metabolism dysfunction is a prominent feature of DM1 and other RNA repeat disorders. Expression of RNAs with expanded CUG repeats is associated with alterations in glucose and lipid metabolism [65–67], mitochondrial dysfunction [67–69] and impaired insulin signalling [70]. These changes involve both functional defects and transcriptomic alterations, including differential gene expression and alternative splicing of genes regulating mitochondrial, lipid and glucose metabolism [71].

A well-characterized metabolic phenotype in DM1 patients is insulin resistance, a feature shared with other repeat-based disorders [72–74]. Insulin resistance is often accompanied by glucose intolerance owing to impaired insulin-mediated glucose homeostasis [75,76]. Mechanistically, the insulin receptor (INSR) transcript undergoes alternative splicing to produce two isoforms: IR-A and IR-B. In DM1, mis-splicing of INSR exon 11, attributed to MBNL1 loss of function, results in increased production of the lower-signalling isoform IR-A [77,78]. Although this MBNL-dependent defect contributes to impaired insulin signalling, transcriptome analyses indicate a broader and more complex regulation of metabolic genes. Indeed, recent studies suggest that energy metabolism dysregulation associated with RNAs with expanded CUGs may result from the disruption of diverse MBNL-independent factors and play an important role in pathogenesis. RNA sequencing (RNA-seq) profiling of DM1 patient liver samples and Mbnl-knockout mice revealed that only a subset of differentially expressed or alternatively spliced genes are shared between the DM1 and MBNL-knockout samples [71]. Importantly, many metabolic defects specific to expanded CUG expression were not explained by MBNL loss alone [71].

Several additional molecular pathways provide insight into these MBNL-independent transcriptome alterations. Expanded CUG/CAG repeat RNAs form highly stable hairpins that resemble canonical Dicer substrates and can be processed by Drosha and Dicer, together with the RNA-induced silencing complex (RISC), generating repeat-derived siRNAs and miRNAs [10,44]. Such small RNAs (siRNAs) have been detected in DM1, HD and SCA1 patient samples as well as mammalian models, where they correlate with silencing of endogenous transcripts carrying complementary repeat sequences [16,63,79], including genes involved in mitochondrial function, lipid turnover and glucose homeostasis [79,80]. Widespread miRNA dysregulation is also observed in DM1 patients and *Drosophila* models [81–83]. While some miRNA changes arise from MBNL loss, others appear MBNL-independent. These likely reflect defects in miRNA maturation mediated by Dicer, or competition between CUG/CAG repeat hairpins and endogenous microRNAs for processing and RISC loading [79]. In addition to small-RNA pathways, other RBPs may contribute to reshaping the metabolic transcriptome. RBFOX1 and RBFOX2 regulate splicing programmes linked to mitochondrial dynamics and nutrient usage [84], and their reduced activity has been linked to exacerbation of metabolic gene mis-splicing and functional defects [85]. Together, these findings indicate that DM1 metabolic dysfunction arises from multiple layers of transcriptomic dysregulation involving both MBNL-dependent and MBNL-independent pathways.

Our research using *Caenorhabditis elegans* DM1 models supports this mechanistic complexity. The DM1 *C. elegans* strains express a green fluorescent protein (GFP) gene with a 3′UTR bearing different CTG repeat lengths in the body-wall muscle cells (figure 2A,B) [86]. Animals expressing expanded CUG repeats (123CUGs) exhibit impaired motility, nuclear foci formation, sequestration of known modifiers, such as Muscleblind (MBL-1, the *C. elegans* orthologue of mammalian MBNL) [63,86] and broad transcriptome changes similar to those observed in DM1 patients. RNA-seq profiling of adult DM1 strains and *mbl-1* mutants revealed limited overlap in transcriptional changes, with widespread alterations independent of MBL-1 loss (figure 3) [87]. Gene ontology (GO) enrichment analysis highlighted disruptions in organic acid metabolism, the tricarboxylic acid (TCA) cycle, triglyceride pathways and fatty acid metabolism—changes robust in DM1 strains but almost absent in *mbl-1* mutants [87]. Notably, transcriptomic profiling of younger DM1 animals at the fourth larval stage (L4) revealed that metabolic disruptions were already present before adulthood [87]. These changes broadened as animals progressed to 2-day-old adults, with an increase in the number of pathways and components affected, suggesting progressive metabolic reprogramming during development.

**Figure 2.**
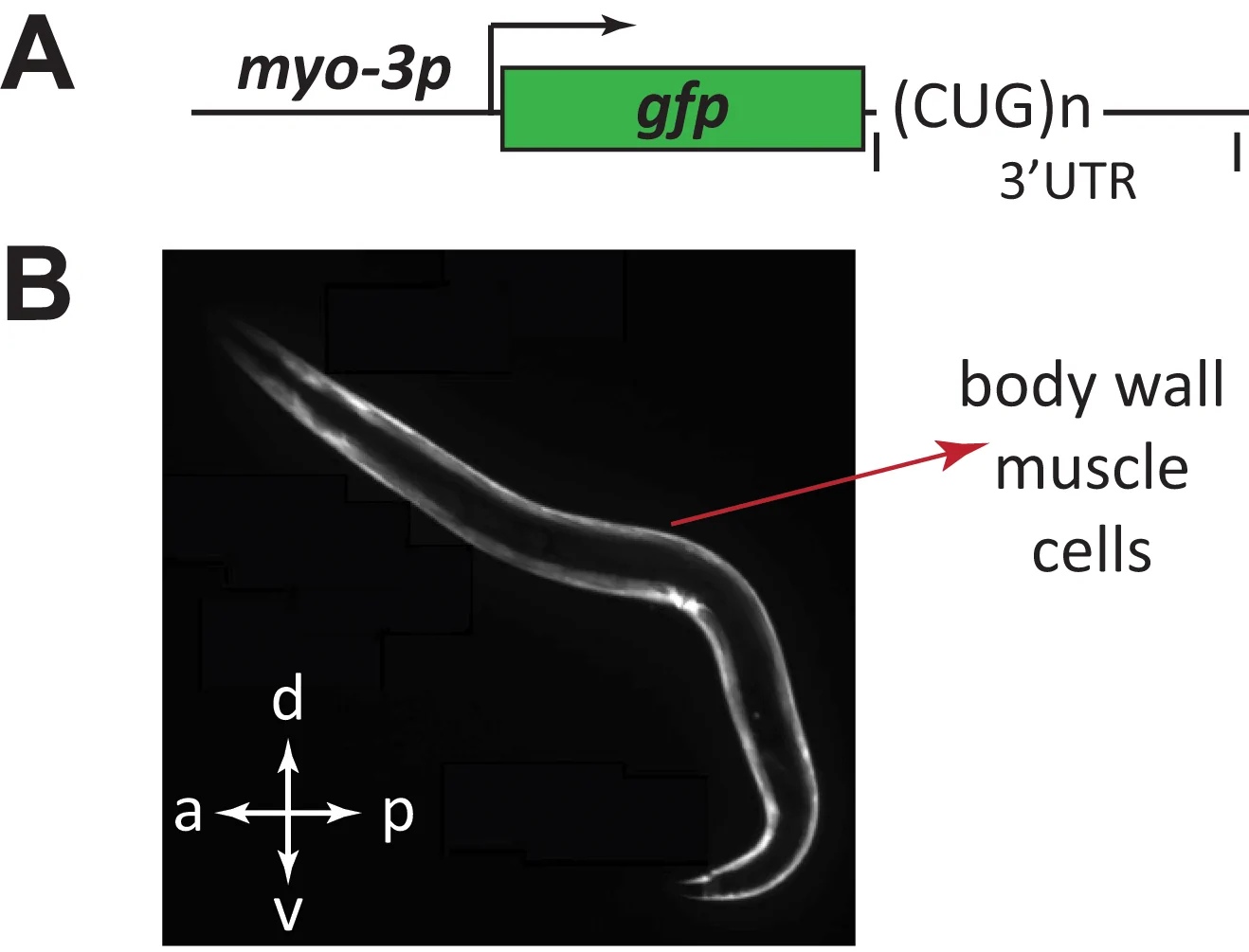
*C. elegans* strains expressing expanded CUGs in body wall muscle cells. (A) Diagram of plasmids used for expression of expanded CUG repeats; (B) Image of *C. elegans* expressing GFP with a 3′UTR bearing 123CUG repeats. The letters in the image indicate the nematode orientation: a, anterior; p, posterior; d, dorsal; v. ventral. Figure adapted from findings described in Garcia *et al.* [86].

**Figure 3.**
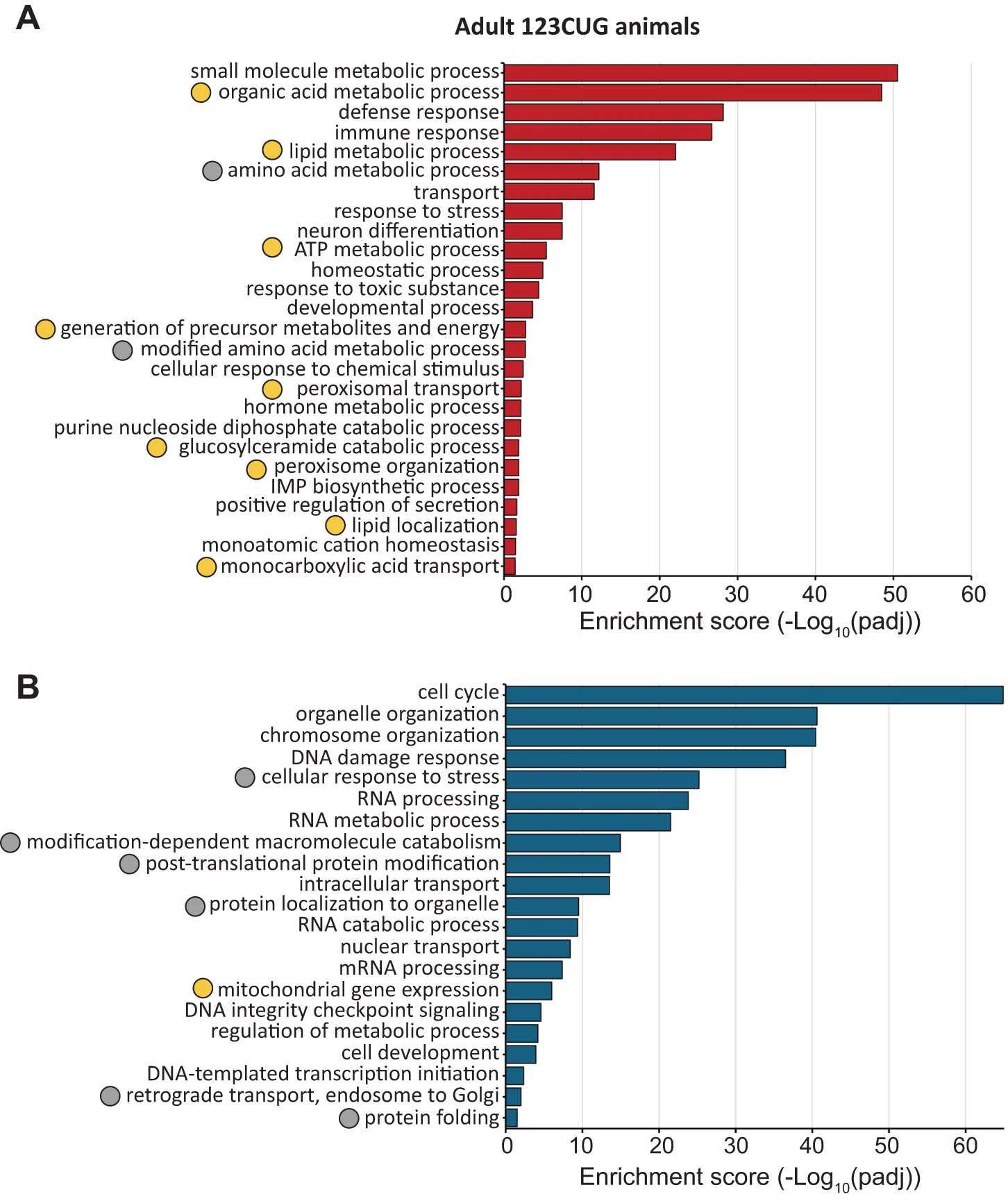
Gene ontology (GO) enrichment analysis of genes altered in 123CUG animals relative to controls (0CUG) as 2-day-old adults. Red bars indicate GO categories enriched among upregulated genes; blue bars indicate GO categories enriched among downregulated genes. Yellow circles and grey circles indicate energy metabolism and proteostasis-related GO categories, respectively. Figure adapted from findings and dataset analysis described in Teixeira *et al.* [87]. The RNA-seq dataset used can be accessed at Gene Expression Omnibus with the accession no. GSE311723.

These findings underscore the multi-layered regulation of energy-related pathways in repeat-based disorders and raise critical questions: How early does energy metabolism disruption begin? Are initial metabolic shifts asymptomatic, and how do they contribute to disease onset and progression? Given that metabolic dysfunction is a hallmark of aging and degenerative disorders, elucidating how these changes are triggered and how they affect organismal fitness, development and stress responses will be essential for understanding pathogenesis and identifying therapeutic targets.

## 5. Mitochondrial disruption in repeat disorders

Mitochondrial dysfunction is a well-documented feature of neuromuscular degenerative disorders, including those caused by repeat expansions [88]. These disorders are frequently described as accelerated aging conditions, where mitochondrial decline has traditionally been considered a secondary consequence of cellular degeneration and toxic processes. However, emerging evidence [87] indicates that mitochondrial impairment is an early and active driver of pathogenesis rather than a late-stage byproduct.

In DM1, clinical observations have consistently revealed metabolic abnormalities that extend beyond insulin and glucose resistance. Early reports documented reductions in Coenzyme Q (CoQ) levels, elevated basal lactate and increased free radical production [89]. CoQ is an essential component of the mitochondrial electron transport chain (ETC) and plays a critical role in oxidative phosphorylation (OxPhos) [90]. More recent studies have further identified alterations in ATP synthesis and the accumulation of structurally abnormal mitochondria in DM1 cells [69,91]. In parallel with these biochemical defects, DM1-derived cells and patient samples exhibit reduced expression of several nuclear-encoded regulators of mitochondrial function and morphology, including TFAM, MFF, OPA1, MFN2 and DRP1 [68,92]. Patient biopsies also show decreased protein levels of multiple mitochondrial-encoded components—such as CO1, ND5, CYB and SLC41A3—many of which participate directly in the ETC [93]. These findings point to widespread dysregulation of both nuclear and mitochondrial gene expression, although the mechanisms driving these alterations, the timing of their emergence during disease progression and how they contribute to disease pathogenesis remain unclear.

Recent studies have begun to address this gap. Garcia-Puga and colleagues [68] used DM1 fibroblasts and blood samples and showed a decrease in OxPhos function, reduced oxygen consumption rates (OCR), diminished ATP production and increased production of reactive oxygen species (ROS). Research from our laboratory, using DM1 *C. elegans* strains, further expanded our understanding of the pathogenic role of mitochondrial disruption, supporting a role for mitochondrial dysfunction as a driver of muscle degeneration [87,94]. Through an unbiased genetic screen for modifiers of RNA repeat toxicity, we identified the CoQ biosynthetic pathway as a suppressor of muscle dysfunction. Inactivation of the *coq-1* gene, the *C. elegans* orthologue of human COQ1, essential for CoQ synthesis, caused severe paralysis and eventual death of animals expressing 123CUGs. Conversely, inactivation of *coq-1* in control animals showed only a mild locomotor impairment [94]. Analysis of the mitochondria in 123CUG animals revealed profound OxPhos disruption, characterized by elevated basal respiration, loss of spare respiratory capacity, decreased membrane potential, increased ROS production and extensive mitochondrial fragmentation. Strikingly, these phenotypes could be ameliorated in 123CUG animals by CoQ supplementation. In addition to reducing mitochondrial disruption, CoQ supplementation also mitigated muscle cell dysfunction, reducing the motility defect of 123CUG animals [94]. Together, these findings link mitochondrial disruption as a central driver in muscle degeneration.

These findings also underscore mitochondrial dysfunction and metabolic reprogramming as early, pathogenic events in RNA repeat disorders that can be reverted. Importantly, they demonstrate that targeting mitochondrial pathways—such as enhancing CoQ availability—can mitigate cellular toxicity and improve tissue function. Given the prevalence of mitochondrial abnormalities across multiple repeat-based disorders, interventions aimed at restoring energy metabolism may offer broad-spectrum therapeutic strategies to complement RNA-targeted approaches. Understanding how toxic RNAs perturb mitochondrial homeostasis will be essential for developing effective treatments for these complex diseases.

## 6. RNA repeat toxicity disrupts cellular proteostasis

Beyond mitochondrial impairment, RNA repeat toxicity exerts widespread effects on cellular homeostasis, including disruption of proteostasis. Expanded repeat RNAs interfere with RBPs, transcriptional regulation, energy metabolism and stress signalling, creating a cascade of dysfunction that compromises protein quality control systems (figure 4). These changes are predicted to be progressive with an impact on processes essential for development, fitness and longevity, and are evident in DM1 transcriptional data, which reveal alterations in protein folding, protein degradation and stress response pathways.

**Figure 4.**
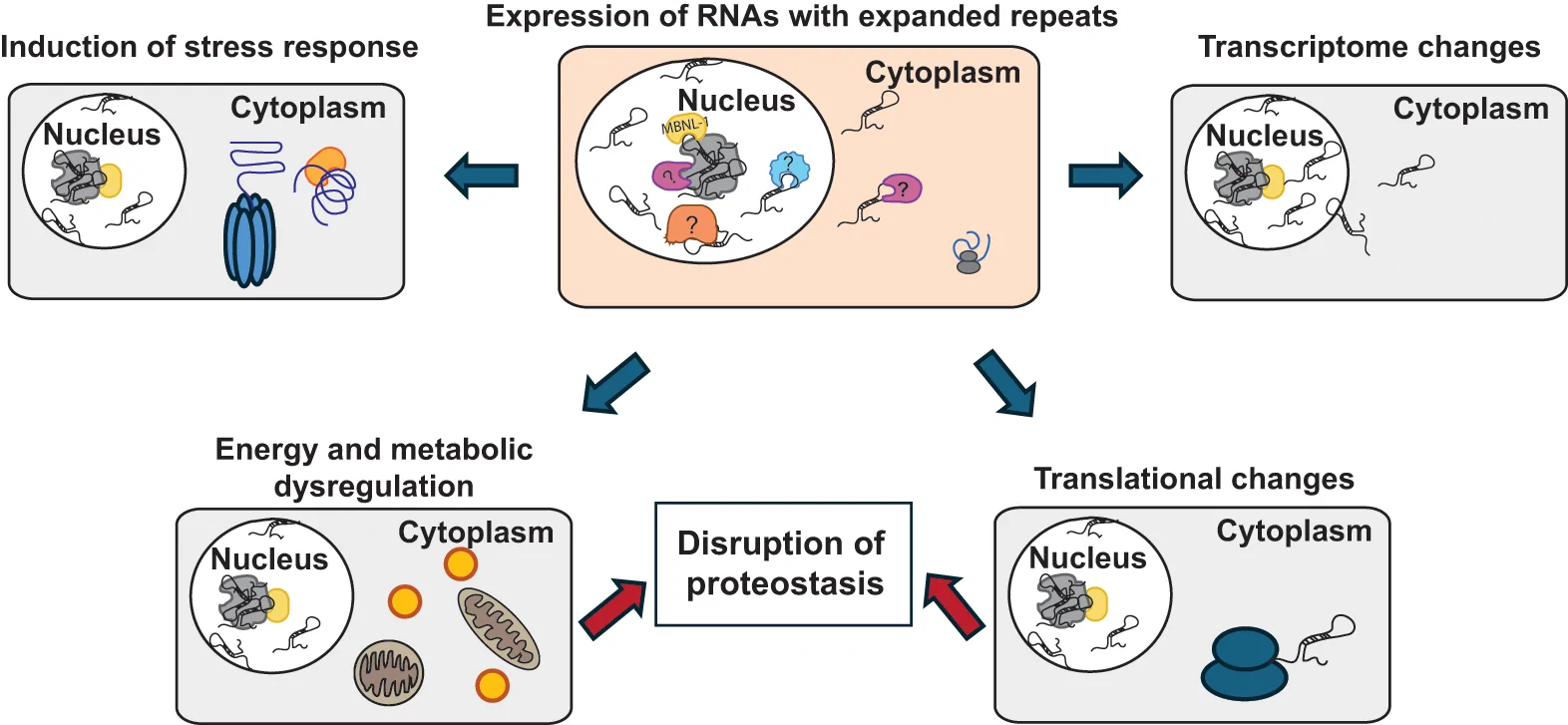
Model of molecular disruptions associated with the expression of RNAs bearing expanded repeats that contribute to disruption of cellular proteostasis.

Mitochondrial dysfunction amplifies this burden on proteostasis. As central regulators of energy production and redox balance, mitochondria influence protein folding and degradation pathways. Their disruption leads to oxidative stress, altered ATP availability and activation of stress-response networks that impair chaperone function, proteasome activity and autophagy. Thus, we predict that mitochondrial and proteostasis defects in repeat disorders are mechanistically intertwined, jointly accelerating cellular decline.

Traditionally, proteostasis failure in repeat-based disorders, such as HD has been attributed to misfolded proteins containing expanded polyQ tracts. However, growing evidence indicates that RNA toxicity also contributes significantly to this process [95–97]. Transcription of expanded repeats in coding regions generates RNAs that form stable secondary structures, sequester RBPs and disrupt splicing and translation. Recent data show that these RNA-mediated perturbations can compound proteotoxic stress by impairing energy homeostasis with the potential to overwhelm protein quality control systems. The result is a synergistic collapse of cellular defences against misfolded proteins and toxic RNAs.

Recognizing RNA toxicity as an additional layer of dysfunction in protein misfolding disorders is critical for a comprehensive understanding of pathogenesis. Therapeutic strategies must therefore address both RNA-mediated and protein-mediated disruptions, targeting mitochondrial and proteostasis pathways alongside direct interventions against toxic repeats. Such integrated approaches hold promise for restoring cellular homeostasis and improving outcomes in repeat expansion disorders.

## Data Availability

The RNA-seq data used to generate the GO term analysis shown in figure 4 is uploaded in the Gene Expression Omnibus (GEO: http://www.ncbi.nlm.nih.gov/geo/) with the GEO accession no.: GSE311723.

## Theme

One contribution of 20 to a Theo Murphy meeting issue ‘ProteostaSys: a systems view of proteostasis’.
